# In Vitro Antiproliferative Activity in Plants of the Genus *Tabebuia*: A Systematic Review

**DOI:** 10.3390/molecules30112315

**Published:** 2025-05-25

**Authors:** Laura Mosquera-Morales, Lina Marcela Orozco, Luz Angela Veloza, Juan Carlos Sepúlveda-Arias

**Affiliations:** 1 Grupo Infección e Inmunidad, Facultad de Ciencias de la Salud, Universidad Tecnológica de Pereira, Pereira 660003, Colombia; lauram2@utp.edu.co; 2 Grupo Polifenoles, Facultad de Tecnologías, Escuela de Química, Universidad Tecnológica de Pereira, Pereira 660003, Colombia; marcely@utp.edu.co (L.M.O.); lveloza@utp.edu.co (L.A.V.)

**Keywords:** *Tabebuia*, cytotoxicity, antiproliferative, apoptosis, angiogenesis, cancer, tumor resistance

## Abstract

The use of plant extracts and the compounds isolated from them for the treatment of cancer is an area of active research, given their therapeutic potential. This work focused on evaluating the literature related to the antiproliferative activity of extracts obtained from plants of the genus *Tabebuia* and molecules isolated in vitro or in vivo. For the search, MeSH and DECS terms were employed in the PubMed, Scopus, and SciELO databases. Research has shown that plant extracts derived from plants of the genus *Tabebuia* exhibit potential applications in the search for new molecules with antiproliferative activity. Among the isolated molecules, the most evaluated correspond to β-lapachone (naphthoquinone); however, molecules with antiproliferative potential belonging to groups such as iridoids, flavonoids, quinones, furanonaphthoquinones, triterpenes, and polysaccharides have also been isolated and reported. Additionally, synthesized molecules have been evaluated on the basis of the modifications made to the structures of molecules isolated from the plant extracts to increase their activity, aiming to develop more potent antitumor agents for future clinical use.

## 1. Introduction

Cancer has prevailed for many years as a disease with high mortality worldwide, currently treated with a variety of tools and specific therapies; however, according to the GLOBOCAN 2020 database, incidence rates (19.3 million new cases) and mortality rates (9.9 million deaths) from cancer continue to be high worldwide. Limiting the search by continents within the database, without discriminating sex and age, Asia has the highest values of incidence and mortality (9,826,539 and 5,464,451 million people, respectively). The Americas ranks third, after Africa and Oceania, with incidence and mortality rates of 1,551,060 and 749,242 million people, respectively. Brazil displays the highest rates, and Paraguay the lowest. Worldwide, female breast cancer is the most commonly diagnosed cancer (11.7% of total cases), followed closely by lung (11.4%), colorectal (10.0%), prostate (7.3%), and stomach (5.6%) cancers [1].

The secondary metabolites of plants have been studied extensively, as they are considered, at the evolutionary level, to be structures developed to defend against pests and insects [2]. Ethnopharmacological knowledge about the use of plants to treat diseases has facilitated the identification of therapeutic agents against various diseases, including cancer. Approximately 75% of the drugs used for cancer treatment are derived from or inspired by plants [3,4]. Tumor cells activate various mechanisms that allow them to survive under stressful conditions and to continue their metabolic and malignant processes. Drugs developed against cancer block mechanisms related to the pathways of angiogenesis or cell apoptosis; however, it has been shown that cancer cells activate alternate mechanisms that allow them to continue their processes, mutating and making chromosomal rearrangements. [2,5] Therefore, the search for new antitumor drugs based on plant extracts, molecules isolated from them, or molecules that complement the action of the drugs currently used, blocking the alternative survival mechanisms of tumor cells, is needed.

The genus *Tabebuia* is the largest in the *Bignoniaceae* family, and it is mainly found in Latin America. The species of this family are mainly ornamental, but they have been widely studied, since molecules with pharmacological activity have been isolated from them [6]. The chemical compounds of this species have been extensively studied [7], with furanonaphthoquinones being the most active group of molecules in the genus *Tabebuia* [5]. Additionally, the presence of naphthoquinones, quinones, benzoic acids, cyclopentene dialdehydes, iridoids, and phenolic glycosides has been detected [7]. The naphthoquinones extracted from the bark of different species of the genus *Tabebuia* are lapachol and β-lapachone [7]. Several studies have determined the biological effects of β-lapachone, including antimicrobial, antiviral, antiparasitic, anti-inflammatory, antiproliferative, and antitumor effects [8,9,10,11]. β-lapachone has been evaluated in various cancers, including adenocarcinoma of the breast; carcinoma of the lung, esophagus, and prostate; colorectal carcinoma; hepatocarcinoma; melanoma; and oral squamous cell carcinoma [9]. However, side effects, such as nausea and vomiting, have been reported in phase I trials [2,7,12]. However, these effects are reduced by treatment with *Tabebuia* extracts, which is attributed to the combined action of all the compounds present, thereby improving tolerability in the body [7]. The therapeutic potential of *Tabebuia* has been attributed to the presence of various bioactive compounds, including flavonoids, alkaloids, terpenes, tannins, and phenolic compounds, which have been extensively studied to elucidate their mechanism of action [13]. Most studies have shown that these phyto-compounds possess high antioxidant capacity and reduce the presence of reactive oxygen species. On this basis, synthetic molecules have been developed with improvements that aim to potentiate the antioxidant effect through structural variations [14].

Grose et al. reported that the genus *Tabebuia* was changed to *Handroanthus* after a taxonomic revision. Some papers no longer mention the genus *Tabebuia* but instead refer to its new name. As a result, the species names have also changed and are now considered synonyms. For example, *Tabebuia heptaphylla* appears in the new taxonomy as *Handroanthus heptaphyllus*, *Tabebuia* alba as *Handroanthus albus*, and *Tabebuia chrysantha* as *Handroanthus chrysanthus* [15]. Although the taxonomic reclassification occurred nearly 20 years ago, the differentiation is still valid; however, the new classification led to considerable taxonomic confusion. The species included in the *Handroanthus* genus can be found at https://www.ipni.org/search?q=handroanthus, accessed on 22 May 2025.

The traditional uses of plants of the genus *Tabebuia* have prompted the study of the biological activities of their extracts and their isolated or synthesized molecules, with a special emphasis on their antiproliferative activity. The genus *Tabebuia* comprises approximately 100 species; its therapeutic potential is widely known within certain communities, and it has been used for centuries in traditional medicine [6]. The inner cortex is known as “Taheebo” or “lapacho”, and it has been traditionally used to treat fever, pain, and arthritis, ulcers, syphilis, gastrointestinal problems, candidiasis, diabetes, prostatitis, and allergies, as well as to prevent various types of cancer [6,7,16,17]. Currently, the dry product of the inner bark of *Tabebuia* is marketed in the form of infusions, pills, and syrups, as is the case with the inner bark of *T. impetiginosa* [7]. The use of plant extracts from the genus *Tabebuia* for cancer treatment began in Brazil in 1960, leading to an increase in the commercialization of the bark of several species, especially *T. impetiginosa* (Mart. Ex DC.). Standl., *T. rosea* (Bertol.) DC., and *T. serratifolia* (Vahl) [6], in various countries in the Americas. The species *Tabebuia chrysantha* is used not only in America but also in India, where it is known as Golden Goddess. The inner bark of species such as *T. impetiginosa*, *T. pallida*, *T. chrysantha*, and *T. rosea* has been used to obtain extracts, and their antioxidant, anti-inflammatory, antimicrobial, and antitumor activities have been demonstrated [11,18,19,20,21,22,23,24]. The species *T. pallida* is native to the Caribbean and is traditionally used in infusions of the leaves and stems to treat fever, pain, cancer, syphilis, inflammation of the tonsils, and malaria. The species *T. aurea* is traditionally used as an infusion or maceration in alcohol, or the bark is chewed to take advantage of its anti-inflammatory activity as part of the treatment for snake bites [25]. The species *Tabebuia avellanedae* Lorentz ex Griseb is commonly known in South America as the “divine tree” because indigenous peoples have traditionally used it because of its versatility and affordability for the treatment of chronic diseases, including cancer [26].

This work aims to explore the anti-proliferative potential of plant extracts obtained from *Tabebuia* species to understand the mechanisms of action of isolated molecules and the potential use of synthesized molecules on the basis of their structure and their promising activities. The potential for plants of the genus *Tabebuia* in the search for new antitumor agents is highlighted, especially given their ability to induce apoptosis in tumor cell lines.

## 2. Results and Discussion

### 2.1. Search Results

**Figure 1:**
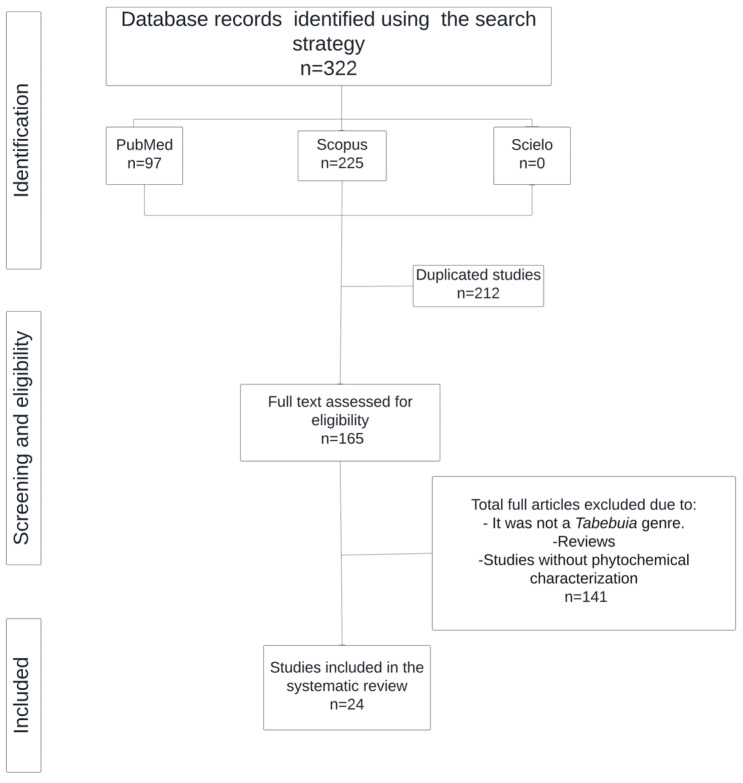
Flow diagram of the study selection process (obtained from the PRISMA 2020 statement, an updated guideline for reporting systematic reviews [27]).

The database search identified a total of 322 articles (PubMed, 97; Scopus, 225; and SciELO, 0). After eliminating duplicates (212 articles), 165 articles were eligible for full-text assessment. Of these, 24 studies were included in this systematic review, and 141 studies were excluded for the reasons presented in Figure 1 and Table 1.

### 2.2. Plant Extracts with Anti-Proliferative Activity

The anti-proliferative and cytotoxic activities of extracts and isolated compounds from different species of the genus *Tabebuia* have been demonstrated in several studies. This section presents the results obtained from studies in which the effects of extracts against various cell lines were evaluated. The extracted data are summarized in Table 2. Despite its anti-proliferative activity, Pires et al. reported that lapachol induced vomiting and nausea in phase I trials. Additionally, β-lapachone has exhibited cytotoxic activity in vitro; however, it has yielded negative in vivo results in mice. When studies of the complete methanolic extract were conducted, the side effects were mitigated. In these studies, Pires et al. reported that the antitumor activity of the extracts resulted from the presence of naphthoquinones, but within the extract, it works in synergy with the other compounds, modulating adverse effects and improving tolerability in the body. This study evaluated the potential of methanolic extracts compared with that of phytopreparations, both of which have high phenolic contents that lead to strong antioxidant activity. Traditionally, *Tabebuia* species have been used in the form of infusions, pills, or syrups for the treatment of arthritis, pain, inflammation of the prostate, fever, dysentery, boils, and ulcers and to prevent different types of cancer. This study highlights the importance of the use of methanol in bioactive compounds since, compared with syrup, methanol extract displays a greater cytotoxic activity (50% inhibitory concentration, IC_50_) against MCF-7 (IC_50_ 110.8 µg/mL), NCI-H460 (IC_50_ 76.7 µg/mL), HeLa (IC_50_ 93.2 µg/mL), and HepG2 (IC_50_ 83.6 µg/mL) cells. Nontumor cells have also been used (primary cells of porcine liver, PLP2) in the assays, and the methanol extracts did not induce cytotoxic activity; thus, Pires et al. reported the selectivity of the extract for tumor cells [7].

Suseela et al. reported that the ethanolic extract of *Tabebuia roseo-alba* can induce early apoptosis in A549 human lung cancer cells; however, this activity was enhanced with the addition of silver nanoparticles, increasing the degree of apoptosis (the IC_50_ values were 300 μg/mL and 200 μg/mL for the ethanolic extracts of *T. roseo-alba* and its AgNPs, respectively). Additionally, Suseela et al. proposed a method for producing nanoparticles from an ethanolic extract, as the secondary metabolites present in plants reduce silver ions, thereby accelerating the nanoparticle synthesis process. Additionally, silver displays both medicinal properties and potential importance in combating cancer. Obtaining nanoparticles through a biological process is less dangerous, more profitable, and less polluting. These findings suggest that the same reducing capacity of the extract that leads to the formation of nanoparticles is responsible for the oxidative stress produced in the cells, which consequently results in cell cycle arrest and apoptosis. Western blot analysis revealed the activation of caspase-3 after treatment with the ethanolic extract, with more pronounced expression after treatment with the nanoparticles. The induced apoptotic pathway is the intrinsic pathway, which involves the activation of caspases 3 and 9, triggered by the release of cytochrome c in response to oxidative stress. The generation of reactive oxygen species leads to alterations in the permeability of the mitochondrial membrane, as well as to DNA fragmentation, which results in the arrest of the cell cycle. A549 cells were arrested in the G0/G1 phase, in the presence of the ethanolic extract obtained from the leaves of *Tabebuia roseo-alba,* and arrested in the G2/M phase after treatment with the silver nanoparticles. The noble metals used in the formation of nanoparticles exhibit antitumor potential, and they act synergistically with the ethanolic extract, which also exhibits antiproliferative and apoptotic activity [13].

El-Hawary et al. [6] designed a profile of the metabolites present in extracts obtained from the leaves and inner bark of five species of *Tabebuia* via metabolomic strategies. They determined how the presence of certain metabolites was related to the cytotoxic activity of the extracts in three cell lines (HepG2, MCF-7, and Caco-2) and identified 40 compounds of different chemical natures which displayed antitumoral potential. A principal component analysis (PCA) was performed to reduce the amount of information by identifying clusters and outliers among all the data collected. The PCA graph displays the total variances for PC1 and PC2 as 42% and 23%, respectively. In the analysis, extracts of the leaves and inner bark were used, and the chemical profiles of both extracts were similar for *T. pulcherrima* and *T. pallida*, but the extracts from the leaves of *T. rosea*, *T. argentea*, *T. guayacan*, and the extract from the inner bark of *T. rosea* were highly dispersed, indicating differences in their chemical profiles. The similarity in composition was related to the cytotoxic activity toward different cell lines. The biological activity results indicated that in *T. rosea* (Bertol.) DC., the stem extract was the most cytotoxic to the HepG2 cancer cell line (IC_50_ of 4.7 µg/mL), whereas the *T. pallida* L. stem was the most active against the MCF-7 cancer cell line (IC_50_ of 6.3 µg/mL). Furthermore, the *T. pulcherrima* stem extract was the most cytotoxic to Caco-2 cells (IC_50_ of 2.6 µg/mL). The molecular correlation analysis highlighted the compounds responsible for the cytotoxic activity against the HepG2, MCF-7, and Caco-2 cell lines. The cytotoxicity in the Caco-2 cell line was not associated with the presence of a particular compound but rather with synergism, and the presence of iridoids and other compounds was determined. This study enabled the detection of molecules directly related to activity in the HepG2 cells and others related to activity in the MCF-7 cells, which are molecules that had not been previously reported; however, the study does not present their characterization or the experiments used to define their mechanism of action [6].

Plant biodiversity has been of great interest in the development of phytopharmaceuticals, and synergistic activity has been established between phytopharmaceuticals and radiotherapy in animal models. Panda et al. [28] reported that phytochemical analysis of the inner bark extract of *T. chrysantha* revealed the presence of quinones, terpenoids, flavonoids, and saponins. The cytotoxic activity of the extract against EAC (Ehrlich ascites carcinoma) cells increased in a concentration-dependent manner, with the highest activity observed (inhibition of growth, IG_50_) at a concentration of 463 µg/mL. Lipid peroxidation and oxidative stress marker tests revealed that the antitumor activity of the extract resulted from an increase in endogenous antioxidant defense systems and the modulation of lipid peroxidation. Treatment with the extract decreased the tumor volume and improved the viable cell count. Panda et al. evaluated the induction of apoptosis concerning the MAPK/ERK activation pathway, since it is deregulated in cancer and is responsible for inhibiting the normal process of apoptosis; for this purpose, they used sEGFR (soluble epidermal growth factor receptor) as a reference. This signaling pathway is particularly important because larger tumors with a greater inflammatory response are typically EGFR-positive. Treatment with the extract promoted the development of early apoptosis, and Western blot analysis did not reveal the activation of the EGFR and ERK proteins, indicating that the MAPK/ERK activation cascade was inhibited. Panda et al. attributed this activity to the presence of naphthoquinones and polyphenols; however, the evaluation of these compounds is required after their isolation [28]. The effects of the methanolic extract of *Tabebuia pallida* on EAC cells were analyzed to elucidate the apoptotic mechanism involved. Rahman et al. [24] reported that apoptosis is the preferred mechanism of cell death in cancer because it does not generate local inflammation. Cancer is characterized by the resistance of tumor cells to apoptosis, a process associated with an imbalance in the expression of antiapoptotic and proapoptotic agents, resulting in uncontrolled cell growth and cancer progression. Among the four methanolic extracts evaluated and obtained from different parts of the plant, i.e., the leaves (TPL), stem bark (TPSB), root bark (TPRB) and flowers (TPF), the highest cytotoxic activity was observed with the extract obtained from the leaves (TPL), which inhibited the growth of tumor cells by 86.2% at an IC_50_ of 120 µg/mL, whereas IC_50_ values of 46, 37.5, and 57 μg/mL were obtained for the TPF, TPSB, and TPRB extracts, respectively [24]. This percentage of inhibition was reduced in the presence of caspase-3 and -8 inhibitors, suggesting that the induction of apoptosis by the TPL extract depends on these caspases. Confirmation of the induction of apoptosis was confirmed by confocal microscopy. mRNA analysis of the proapoptotic (p53, PARP-1, and Bax) and antiapoptotic genes (NFk-B, Bcl-2 and Bcl-xl) revealed that the TPL extract maintained equilibrium, promoting apoptosis in EAC cells. A compositional analysis of TPL (highly active part) was performed via LC–PDA–MS/MS to identify which phytochemicals are responsible for its anticancer activity. The apoptotic activity was attributed to quercetin-3-glucoside (major compound), pelargonidin-3-O-glucoside, and/or lapachol derivatives.

Rahma et al. reported reliable parameters to judge the efficacy of antitumor drugs, including a reduction in tumor cell growth, a decrease in tumor weight, a decrease in white blood cell count, and prolongation of cell survival. TPL suppressed cell growth, decreased tumor size, and increased red blood cell levels, which is why Rahman et al. reported that TPL has a protective effect on cells of the hematopoietic system [24].

The extracts of the leaves and inner bark of *T. rosea* were analyzed by Jimenez-Gonzalez et al. [11] to determine their antioxidant, anti-inflammatory, and antiproliferative activities. All the extracts contained quinones, anthrones, coumarins, terpenes, steroids, and sesquiterpene lactones; however, phenols and flavonoids were observed only in the ethyl acetate extract. This study reports values of 50% cytotoxic concentration (CC_50_), 50% inhibitory concentration (IC_50_), and even selectivity index (SI) results, which are not reported in most of the reviewed publications. Jimenez-Gonzalez et al. reported, for the first time, the antiproliferative activity of extracts obtained from the inner bark of *T. rosea*, and the highest activity concerning the selectivity index was induced by the chloroform extract obtained from the inner bark for the cell lines evaluated (HepG2, 21.1 ± 1.4 μg/mL, SI = 5.50; B16F10, 36.4 ± 1.7 μg/mL, SI = 3.18; MCF7, 45.5 ± 1.2 μg/mL, SI = 2.55; and HeLa, 57.6 ± 1.2 μg/mL, SI = 2.01 [11]. Compared with the different cell lines evaluated, the chloroform extract of leaves also displayed the best IC50 values: MCF-7, 5.0 ± 1.2 μg/mL; HepG2, 17.3 ± 1.3 μg/mL; B16F10, 17.6 ± 1.3 μg/mL; and HeLa, 24.7 ± 1.4 μg/mL [11]. The antiproliferative potential of *Tabebuia* extracts has been widely demonstrated. In this context, Guerrero-Pepinosa et al. conducted a study evaluating the effect of the *n-butanol* extract obtained from the inner bark of *Tabebuia rosea*, along with two isolated iridoids (catalposide and specioside), in synergistic combination with apicidin (APC), a histone deacetylase inhibitor (HDACi). The study, conducted on human monocytic leukemia cells (THP-1), demonstrated the dose-dependent cytotoxicity of the evaluated compounds, with IC_50_ values of 44.7, 43.9, and 40.3 μg/mL for the n-butanol extract, catalposide, and specioside, respectively, at 24 h [22].

The results revealed the induction of apoptosis through the intrinsic mitochondrial pathway, as evidenced by an increase in *Bax* expression, a decrease in *Bcl-2*, and the loss of the mitochondrial membrane potential. This study highlights the role of the p38 protein, a recognized target in pharmaceutical research, whose phosphorylation is promoted by APC pretreatment. APC also facilitates the expression of proapoptotic genes such as *Bax*, which are typically repressed in cancer cells, suggesting a synergistic epigenetic effect that enhances the cytotoxic action of iridoids.

In addition, cell cycle arrest at the G0/G1 phase was associated with p38 activation, further supporting its involvement in cell cycle regulation. Overall, this study proposes a combined therapeutic strategy in which natural extracts with antiproliferative activity enhance their efficacy through interaction with epigenetic modulators such as APC [22].

In the study by Muruganandham et al., the cytotoxicity of silver nanoparticles (AgNPs) synthesized using an aqueous extract of *Tabebuia rosea* seeds was evaluated. Although this article focuses primarily on the physicochemical characterization of the nanoparticles and their antibacterial and antioxidant properties, it also examines their biological effects on cells via an MTT-based cell viability assay [29]. The cell line used was L929, which corresponds to murine fibroblasts. Treatment with AgNPs dose-dependently inhibited cell growth, resulting in a 17.12% reduction at 100 µg/mL and an IC_50_ value of 45 µg/mL. Morphological changes consistent with apoptotic processes, such as cell retraction and detachment, were also observed. However, it is essential to note that the study did not include tumor cell lines, which makes it impossible to claim specific antiproliferative activity against cancer cells. Instead, the current findings reveal a potential toxic effect on healthy cells, highlighting the need for comparative assays with tumor cells, along with selectivity studies. Despite its promise as a nanoformulation, this work leaves open the possibility of its direct applicability in cancer therapies, as the specificity of the compound against malignant cells has yet to be evaluated [29].

The reviewed studies confirm the antiproliferative and cytotoxic potential of extracts obtained from plants of the *Tabebuia* genus. The antitumor effects observed depend not only on the specific metabolites present in the extracts but also on the synergy between these compounds. While adverse effects have been reported for isolated molecules, the use of complete methanolic extracts appears to mitigate these effects, highlighting the importance of compound combinations in modulating toxicity and improving tolerability.

Suseela et al. reported that the incorporation of silver nanoparticles enhances the cytotoxic activity of the ethanolic extract of *Tabebuia roseo-alba*. These findings suggest that the reducing capacity of plant metabolites not only contributes to nanoparticle formation but also increases oxidative stress and apoptosis in tumor cells [13].

El-Hawary et al. [6] identified 40 compounds with antitumor potential through metabolomic analysis and correlated their cytotoxicity with the chemical composition of the extracts. Specifically, the stem extract of *T. rosea* demonstrated the greatest cytotoxicity against the HepG2 cell line. Moreover, *T. pallida* exhibited greater activity against the MCF-7 cell line, suggesting selectivity in the action of certain compounds.

Additionally, Panda et al. [28] demonstrated that extracts of *T. chrysantha* induce apoptosis by inhibiting the MAPK/ERK activation pathway, a key mechanism in the proliferation and survival of tumor cells. These findings support the hypothesis that *Tabebuia* compounds may act by blocking specific signaling pathways associated with tumor growth. This finding is further supported by Rahman et al. [24], who reported that the methanolic extract of *T. pallida* not only induces apoptosis in Ehrlich ascitic carcinoma (EAC) cells but also has a protective effect on the hematopoietic system, reducing tumor size and improving cell survival.

Finally, Jiménez-González et al. [11] highlighted the importance of IC_50_ values and selectivity indices, showing that *T. rosea* extracts, particularly those obtained with chloroform, exhibit strong antiproliferative activity. This suggests that the polarity of the extraction solvent has a significant effect on the bioactivity of the extract.

### 2.3. Isolated Molecules with Anti-Proliferative Activity

Owing to the traditional use of different *Tabebuia* species, the study of the activity of isolated compounds has generated significant interest. This section highlights the results of twelve studies, and the extracted data are summarized in Table 3. The findings include various isolated molecules, such as iridoids [2], flavonoids [5], quinones [8,9,10,30,31,32], naphthoquinones [33], furanonaphthoquinones [26], triterpenes [34], and polysaccharides [35].

Some of the secondary metabolites in *Tabebuia* that have been evaluated for their antiproliferative activity include iridoids. Their structural analysis and their ability to form stable compounds were investigated by extracting iridoids from *T. argentea* leaves [2].

Del Piaz et al. [2] evaluated the ability of iridoids isolated from *T. argentea* to inhibit the ATPase activity of HSP90 upon stable coupling. For this purpose, surface plasmon resonance (SPR) was employed, which enabled the determination of the coupling between the iridoid and the chaperone. This led to satisfactory results for one of the eight iridoids isolated from *T. argentea* (argenteoside A), which inhibited the activity of the HSP90 chaperone α [2].

Inhibition of the ATPase activity of HSP90 can lead to the misfolding of proteins assisted by chaperones, degradation, or the dysfunctional formation of proteins. For this reason, there is interest in investigating the inhibition of HSP90 as an additional mechanism for cancer treatments; however, further studies are needed to determine the effect of HSP90 inhibition on the folding of proteins essential for cell function. In this study, the analysis of the antiproliferative activity of the evaluated compounds was not adequately reported.

**Figure 2:**
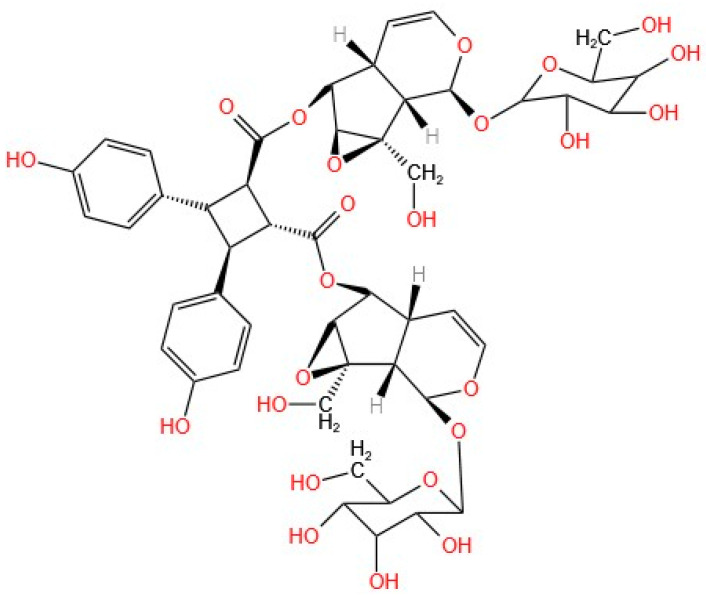
Chemical structures of the iridoid, isolated Compound **A** (argenteoside A). Figure created with KingDraw, 2018–2025.

Argenteoside A (Figure 2) was structurally characterized via various techniques, including the use of a C9-type iridoid, which is characterized by a cyclopentane ring in its structure. In addition to argenteoside A, seven other iridoids have been isolated from *T. argentea*. This compound exhibited stable coupling and inhibition of the ATPase activity of the HSP90 chaperone, displaying a behavior very similar to that of the positive control, i.e., radicicol, a natural compound that has been previously determined to bind to and inhibit the HSP90 protein. The stability of the compound generated by the coupling was determined by measuring K_D_ (equilibrium dissociation constant) and K_d_ (kinetic dissociation constant). The low values for K_D_ and K_d_ demonstrate that the interaction of the chaperone with argenteoside A does not require high concentrations or extended times for the coupling to take place, which translates into good interaction between the iridoid and the chaperone and the stability of the resulting compound [2].

Del Piaz et al. reported that only C9-type iridoids can interact with chaperones, resulting from their structural characteristics, as the p-hydroxybenzoyl group present in argenteoside A is decisive for this interaction. The glycosides present in the structure and the multiple binding sites in the central part of the C9-type iridoid improve the structure–activity relationship of the compound [2].

Based on the strong inhibition of ATPase activity resulting from the binding of argenteoside A and the chaperone, a coupling analysis was performed to evaluate the inhibition of the chaperone. For this purpose, a thermally induced aggregation citrate synthase test was conducted. Radicicol was used as a positive control for this test. The results demonstrate that the inhibition of the chaperone is attributed to stable coupling with Compound **A**, since the behavior is the same as that shown without the presence of the chaperone. [2].

An antiproliferative evaluation was conducted using HeLa cells to assess the effects of the iridoid on the proteins HSP90 and HSP70 (IC_50_ > 100 μM). After 24 h, the analysis revealed low regulation of several proteins involved in survival, structure, and cell proliferation; these are client proteins in the activity of the chaperone. Structural analyses of the compounds bound to the protein demonstrated the high affinity of the iridoid for the chaperone, providing insight into their stability. Previously, the structural characteristics of C9-type iridoids were reported to be responsible for good coupling. The glycosidic groups of the iridoid backbone allow for binding to the protein via the amino acids Asp93 and Thr184, as do the phenolic groups. In addition, it was demonstrated that Compound **A** binds to the N-terminal end, where the ATPase activity of the protein is located, as confirmed by proteolysis–mass spectrometry, which revealed the flexible and weak regions of the protein after binding.

The structural study of iridoids and the analysis of their anti-proliferative activity provide clear evidence that they are candidates for oncological therapy; however, an in-depth analysis of the selectivity index, which was not conducted in this study, is necessary.

Moreover, researchers have reported high contents of flavones in 16 species of the *Bignoniaceae* family. A previous analysis by Panda et al. [5] revealed that the flavonoid content in the methanolic extract of *Tabebuia chrysantha* was 118.4 mg QE/g. This led to a new analysis to determine the activity of the isolated compound. The development of angiogenesis requires the participation of proteins such as VEGF and MMP9, which are deregulated in tumor cells. Panda et al. suggested that one of the mechanisms of action of anticancer drugs is VEGF receptor blockade; however, resistant cells activate the STAT3-MMP9 mechanism, which promotes angiogenesis. The extraction of a flavonoid (trimethoxyflavone, TMF) from *T. crysantha* and its characterization suggest that VEGF mediates the inhibition of STAT3-MMP9 signaling, which is associated with the suppression of angiogenesis and the proliferation/growth of vascular tumors (angiosarcoma). Natural polyphenols are antioxidants, and the determination of the antioxidant activity of TMF strongly depends on their concentration, which inhibits 1,1-diphenyl-2-picryl-hydrazyl (DPPH), nitric oxide (NO), and hydroxyl radical (OH) scavenging activity [5].

The antioxidant activity of TMF led to a new analysis of its ability to inhibit ERK and reduce p-STAT3, thereby affecting the activation of the angiogenesis pathway. Free radicals are responsible for cell damage, which can lead to diseases such as cancer. In the presence of free radicals, mitochondria increase the production of proteins such as ERK and STAT3 to survive oxidative stress and induce angiogenesis [5].

Panda et al. conducted an ex vivo chorioallantoic membrane (CAM) model assay in which cell proliferation was induced and the cells were exposed to different concentrations of TMF, demonstrating the suppression of angiogenesis. Immunoblotting revealed that TMF inhibits the expression of proteins responsible for the angiogenesis pathway in cells and that ERK is involved in the downregulation of STAT3. qRT–PCR analysis revealed low levels of STAT3 and MMP9 protein mRNA after treatment with TMF, suggesting that this flavonoid suppresses angiogenesis [5].

β-lapachone is a natural quinone extracted from the bark of lapacho trees. It has been extensively studied, and its anti-inflammatory and anti-proliferative activities have been demonstrated. Jeon et al. reported that treatment of different tumor cell lines with β-lapachone prevents cell proliferation, inhibiting the action of topoisomerases that can repress telomerase activity. In addition, β-lapachone induces the downregulation of Sp1 in oral squamous cell carcinoma, which leads to the induction of apoptosis. Sp1 is a crucial transcription factor that regulates the expression of proteins essential for fundamental cellular processes, including proliferation, differentiation, and apoptosis. Sp1 is deregulated in tumor cells and plays a significant role in tumorigenesis [30]. The negative regulatory effect of Sp1 was analyzed in HN22 and HSC4 cells treated with β-lapachone, and the results indicated that Sp1 regulates the expression of proteins involved in the apoptotic process. In these cell lines, negatively regulating Sp1 increases the expression of p21 and p27 proteins, which halt the cell cycle, and decreases the expression of cyclin D1 and survivin, which regulate the cell cycle. The downregulation of cyclin D1 was demonstrated via fluorescence-activated cell sorting (FACS) and 4′,6-diamidino-2-phenylindole (DAPI) analysis, where condensation of the DNA and cells in the sub-G1 phase was observed, indicating the initiation of the apoptosis process. Western blot analysis was performed on lysed HN22 and HSC4 cells after treatment with 0, 1, 2, and 3 µM of β-lapachone for 48 h. The results demonstrated that the apoptotic pathway triggered by β-lapachone is induced by the activation of caspase-3 and PARP [30].

In the treatment of oral squamous cell carcinoma with β-lapachone, β-lapachone negatively regulates Sp1, leading to the activation of proapoptotic proteins, thereby inducing apoptosis. Bang et al. reported that the potential of β-lapachone as an antitumor agent in the treatment of human malignant melanoma was evaluated in two human malignant melanoma cell lines, G361 and SK-MEL-28. The viability of the G361 cells was 56.5 ± 0.02, 50.9 ± 0.01, and 50.5 ± 0.01 after treatment with 1, 2, and 3 μM of β-lapachone, respectively. The viability of the SK-MEL-28 cells was 74.5 ± 0.03, 44.1 ± 0.03, and 37.0 ± 0.02 after treatment with 1, 2, and 3 μM of β-lapachone, respectively. DAPI analysis revealed rounded cells and chromatin condensation, characteristic of cell arrest in the sub-G1 phase, which was due to the inhibition of cyclin D1 by the cell cycle negative regulator proteins p27 and p21 [8].

Quinone was shown to induce apoptosis via the intrinsic pathway by activating caspase-3 and cleaving PARP, while decreasing Bcl-2 and increasing Bax. These results, coupled with the analysis of apoptosis via annexin V and the assessment of the mitochondrial membrane potential, confirm that β-catenin induces apoptosis by negatively regulating Sp1 and altering the expression of various proteins that enable tumor cells to survive and counteract the apoptotic process.

Bax, a proapoptotic protein, is related to the release of cytochrome c from the inner mitochondrial membrane, which is why, in a membrane potential analysis, it is diminished in cells undergoing an apoptotic process.

In summary, the Sp1 protein regulates the expression of genes involved in cellular functions essential for survival; however, it is overexpressed and deregulated in diseases such as cancer, which is why cancer cells exhibit continuous growth. Decreasing the expression of Sp1 in cancer cells induces apoptosis by negatively regulating antiapoptotic proteins and is associated with the cell cycle. β-lapachone reduces the expression of Sp1, leading to the downregulation of its associated proteins; thus, β-lapachone arrests the cell cycle and induces apoptosis in human malignant melanoma [8].

In previous studies, β-lapachone has been shown to induce apoptosis in various types of cancer. In this case, Kee et al. reported that, in addition to inhibiting the proliferation of CT26 colorectal cancer cells, the pathway that activates β-lapachone was determined to induce apoptosis, arrest the cell cycle, and inhibit lung metastasis in colorectal cancer [31], indicating that the metastasis process requires several factors, including damage to the extracellular matrix (EMC), which allows cells to travel from the primary tumor to target organs; additionally, colorectal cancer metastasizes to the lungs, liver, peritoneum, and lymph nodes. In this process, MMP metalloproteinases play a key role, as they exhibit deregulated activity in tumor cells and are responsible for EMC degradation. Treatment with 0–1 and 10 µM of β-lapachone resulted in decreases in the levels of MMP-2 and MMP-9 and increases in the level of the TIMP tissue inhibitor, which regulates the activity of metalloproteinases. During the metastasis process, epithelial–mesenchymal transition (EMT) occurs, during which morphological changes transpire, resulting in the transformation of epithelial cells into mesenchymal cells. Consequently, cell adhesion decreases, facilitating metastasis. During the EMT process, the expression of N-cadherin, the protein responsible for cell adhesion, decreases, whereas other EMT markers, such as E-cadherin, vimentin, and Snail, which are involved in the loss of cell adhesion and the acquisition of migratory properties, increase. Additionally, this process involves evasion of the apoptosis pathway. β-lapachone had an inhibitory effect on EMT markers. Treatment of CT26 cells with 100 nM of β-lapachone decreased E-cadherin, vimentin, and Snail but increased N-cadherin, indicating that β-lapachone inhibits metastasis by increasing the adherence of epithelial cells and preventing their morphological and characteristic changes to the mesenchymal cells.

Kee et al. also conducted an analysis via annexin V, and the results revealed that β-lapachone can induce apoptosis via both intrinsic and extrinsic pathways by initially activating caspase-8, which in turn induces the expression of caspase-3. Western blot analysis revealed that treatment with β-lapachone increases the expression of Bax, thereby suppressing the activity of Bcl-2 and Bcl-xl, while also increasing the expression of cleaved caspases 3 and 9. These findings suggest that the expression of caspase-8 clears the Bcl-2 protein and initiates the process of apoptosis. The inhibition of Bcl-2 increases the expression of Bax, which initiates apoptosis by releasing cytochrome c through the permeabilization of the mitochondrial membrane. This release activates caspases 3 and 9, triggering apoptosis via the intrinsic pathway. Consequently, β-lapachone induces apoptosis through both routes, and this process depends on the time and concentration used in β-lapachone treatment [31].

In several publications, the anticancer activity and pharmacological potential of β-lapachone have been demonstrated. Dias et al. also analyzed two iodine derivatives of β-lapachone. All analyses were performed with doxorubicin used as a positive control. Thirteen tumor cell lines were used for the cytotoxicity analysis, which revealed different values for β-lapachone and its two derivatives [9]. The SI analysis was performed against three noncancerous cell lines (HaCat, MRC5, and PBMC) and was calculated using the following formula: SI = IC_50_ [noncancerous cells]/IC_50_ [cancerous cells]. The three compounds evaluated (β-lapachone, 3-I-α-lapachone, and 3-I-β-lapachone) showed better selectivity for cancer cells, which suggests a benefit of the treatment by reducing systemic toxicity and improving the quality of life of patients. To confirm the cytotoxic effects of the compounds, a three-dimensional in vitro model was used to evaluate factors such as the tumor microenvironment, with IC_50_ values of 9.8 μM for β-lapachone, 8.1 μM for 3-I-α-lapachone, 2.3 μM for 3-I-β-lapachone, and 43.5 μM for doxorubicin. These values were lower than those obtained in the two-dimensional model, which can be attributed to the action of the extracellular matrix, which prevents the drug from entering the cell. After treatment with these compounds, cell morphology analysis revealed that they are permeable, making them good candidates for anticancer drugs [9].

β-lapachone has been shown to have antiproliferative effects by stopping the cell cycle. In this case, through flow cytometry analysis and measurement of the amount of internucleosomal DNA, it was concluded from the variation in concentrations and exposure time that β-lapachone and its derivatives can arrest the cell cycle in the G2/M phase in 40.6% and 41.5% of HSC3 cells after treatment with β-lapachone and 3-iodo-α-lapachone, respectively, at a concentration of 1 μM. Long-term exposure to HSC3 and variations in the concentrations of β-lapachone and its derivatives were associated with decreases in cell volume, chromatin condensation, the presence of apoptotic bodies, and nuclear fragmentation. β-lapachone and its iodine derivatives reduce the membrane potential and increase the production of reactive oxygen species (ROS). In addition, the activation of caspases, Bax, and the extrinsic pathway receptor FAS, as well as the overexpression of the p21 gene, were demonstrated. Therefore, the mechanism of action of β-lapachone and its derivatives is the induction of apoptosis via both routes [9].

β-lapachone has been shown to exhibit potent anticancer activity in lung cancer cells, such as A549 cells; however, cell lines such as CL1-1 and CL1-5 also display low sensitivity to β-lapachone treatment (at a concentration of 5 μM) [32]. Kung et al. highlighted the importance of NQO1, an oxidoreductase that interferes with cellular metabolism and enhances the cytotoxic effect of β-lapachone, on cancer cells. In this way, combination therapy is necessary to increase the expression of NQO1 in cancer cells that are not responsive to treatment with β-lapachone. Some treatments, such as cisplatin, heat shock, or radiation, improve the expression of NQO1, but they are not selective and affect healthy cells as well; for this reason, this analysis was carried out to determine whether sulindac and its metabolites (an anti-inflammatory drug with chemoprotective effects and chemotherapeutics) could increase the expression of NQO1 in cell lines other than A549 [32].

NQO1 reduces β-lapachone to hydroquinone; this process can generate oxidative stress, polarize the mitochondrial membrane, permeabilize it, and release cytochrome c protein, causing cell death. In this analysis, treatment with β-lapachone did not affect H_2_O_2_ levels, but the MMP values decreased dramatically, indicating that ROS other than H_2_O_2_ were generated. To determine whether the cytotoxicity induced by β-lapachone was caused by apoptosis, the use of anexin V analysis revealed that the number of cells in the G0/G1 phase increased over time. The mechanism of action of β-lapachone was also analyzed in relation to the expression of signaling proteins. The expression of JNK, which is regulated by death signals, was increased, whereas the expression of ERK, PI3K, and AKT, which are signals of cell survival, was decreased. The intracellular calcium concentration is a crucial measure, as it serves as a key messenger in cells and plays a vital role in various signaling pathways, including apoptosis. β-lapachone was shown to increase calcium levels in CL1-1 and CL1-5 [32].

All the above findings reveal the mechanism of action of β-lapachone in lung cancer cell lines; however, these cell lines exhibit low sensitivity to quinone treatment due to their low activity levels and the expression of NQO1. To determine whether NQO1 is a regulator of β-lapachone-induced cell death, the NQO1 inhibitor dicoumarol was used. The results revealed that this inhibitor reduces the cytotoxic activity of β-lapachone by 67% in CL1-1 cells and 77% in CL1-5 cells. In this study, the authors also investigated whether the anti-inflammatory agent sulindac and its metabolites could enhance the cytotoxic effect of β-lapachone in CL1-1 and CL1-5 cells, which exhibit low NQO1 expression. To determine whether sulindac regulates the expression of multiple oxidative enzymes, such as NQO1, CL1-1, and CL1-5, the cells were pretreated with sulindac or its metabolites and β-lapachone at sulindac concentrations of 100 µM and 200 µM for different durations of exposure. The results demonstrated more significant cell death in the CL1-5 cells, which presented lower NQO1 expression, suggesting that synergistic treatment with sulindac and β-lapachone enhanced the therapeutic effects of the latter in the treatment of lung cancer [32].

The NQO1 enzyme has been studied as a therapeutic target in cancer treatments and is considered a determining factor in the cytotoxic process of β-lapachone. The low sensitivity of cancer cells to β-lapachone treatment led to an increase in NQO1 expression. In parallel, treatments such as ionizing radiation, hyperthermia, and cisplatin synergistically interact with β-lapachone, as they induce the expression of NQO1, but as mentioned above, these treatments can damage healthy cells; consequently, the need is to identify treatments that act synergistically to improve the therapeutic effect, reduce the dose, increase the efficacy, and minimize the toxicity in normal cells and the resistance of tumor cells. NQO1 is highly expressed in breast, colon, and lung tumors, suggesting that treatment with β-lapachone should be more specifically employed [32].

Lamberti et al. reported that ROS generation at the cellular level precedes the increase in NQO1 expression and that modulation of the redox state enhances the sensitivity of cancer cells to β-lapachone (IC_50_ 2.9 ± 0.03 μM); thus, they proposed a synergistic treatment involving photodynamic therapy (PDT) and β-lapachone. To apply PDT, ME-ALA (5-aminolevulinate methyl ester) was used, and the process was carried out in two steps: first, ME-ALA was administered, and then, it was activated with light, which selectively destroys cancer cells by generating ROS. These ROS molecules lead to the expression of NQO1, increasing the sensitivity to β-lapachone. The results of the cell viability analysis did not reveal significant differences when PDT was applied alone. Synergistic therapy yielded optimal results only when NQO1 was previously activated for 24 h with ME-ALA, followed by treatment with β-lapachone. The IC_50_ values of β-lapachone combined with ME-ALA/PDT (0.5 mM + 1.5 J/cm^2^) were 3.14 ± 0.01 μM and 2.48 ± 0.01 μM, respectively [10].

Various combinations of synergistic treatments were performed, and CI (combination index) isobologram and DRI (dose-reduction index) tests were performed. The results of the isobologram showed synergy for combinations 6 and 9 (combination 6: 2 J/cm^2^ of 0.5 mM ME-ALA and 2.3 μM β-lapachone; combination 9: 2 J/cm^2^ of 0.5 mM ME-ALA and 2.8 μM β-lapachone).

The data points of the experimental combination therapy are plotted well below the expected additive line when the MCF7-c3 cells were photosensitized with ME-ALA/PDT at 2 J/cm^2^ and then treated with 2.3 μM β-lapachone (CI = 0, 71, Combination 1) or 2.8 μM β-lapachone (CI = 0.85, Combination 9), indicating moderate synergism. The authors concluded that the increase in NQO1 activity 24 h after 2J/cm^2^ ME-ALA/PDT sensitized the cells to β-lapachone. Interestingly, when the MCF-7c3 cells were subjected to sublethal doses (0.5 mM + 1 J/cm^2^) of ME-ALA/PDT, they acquired resistance to subsequent chemotherapy treatment [10].

**Figure 3:**
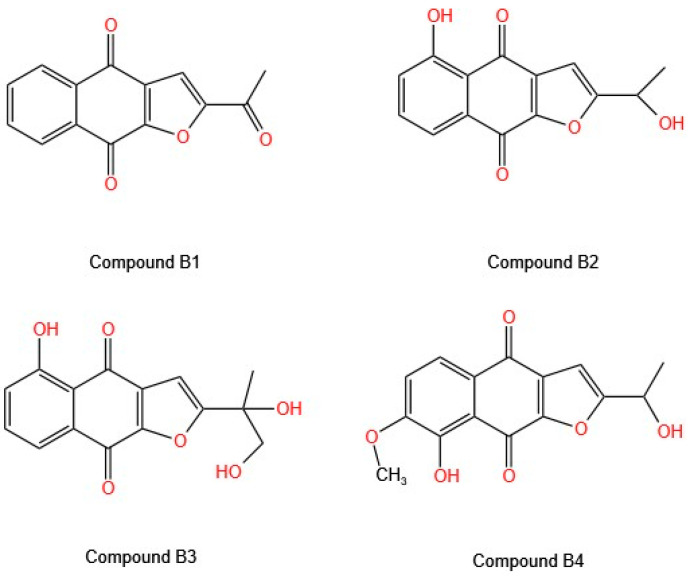
Chemical structures of furanonaphthoquinones and isolated Compounds **B1**–**B4**. Figure created with KingDraw, 2018–2025.

The cytotoxic capacity of four compounds of natural furanonaphthoquinones isolated from *T. Avellanedae* (Figure 3) was evaluated against three cell lines (A549, MCF-7, and SiHa) [26]. In the MTT assay, Compounds **B1**–**B3** showed dose-dependent cytotoxic activity, whereas Compound **B4** showed no cytotoxic activity, even when its concentration was increased. Subsequent analyses focused on Compounds **B2** and **B3**, both of which have a hydroxyl group at position 5, which appears to be related to their cytotoxic capacity. The arrest of the cell cycle is a crucial process in cancer treatment, and as evidenced in studies evaluating isolated molecules, many of them can halt the cell cycle. Zhang et al. [26] conducted a study in which the treatment of A549 cells with Compounds **B2** and **B3** increased the number of cells in the G2/M and S phases and decreased the number of cells in the G1 phase, at the following levels: 2.3 times and 3.3 times more cells in the G2/M phase with Compounds **B2** and **B3**, respectively; 2.8 times and 1.9 times more cells in the S phase with Compounds **B2** and **B3**, respectively; and a decrease in the number of cells in the G1 phase of 3.3 times and 2.5 times with Compounds **B2** and **B3**, respectively. The progression of the cell cycle involves the intervention of a signaling cascade in which a variety of proteins, such as cyclins A and B, are increased in cells that are prepared for mitosis. A549 cells treated with Compounds **B2** and **B3** presented a decrease in the expression of cyclins A and B1, which explains the mechanism of action of these compounds, as they prevent the progression of the cell cycle by arresting it in the G2/M phase. The expression of the D1 protein, an essential signaling protein involved in the cell cycle, decreased after 36 h of exposure [26].

When the induction of apoptosis was evaluated, Compounds **B2** and **B3** demonstrated apoptotic rates of 17% and 5%, respectively, after 36 h of exposure, and 23% and 27%, respectively, after 48 h of exposure. Various proapoptotic and antiapoptotic genes intervene in the apoptotic process, and their activation is triggered by the activated pathway to induce cell death. The expression of various genes was evaluated in A549 cells treated with Compounds **B2** and **B3**; the mRNA level of *P53*, a tumor suppressor gene, increased after 6–12 h of treatment. Additionally, upregulation of the gene and the proapoptotic protein Bax was observed after 36 h of treatment with Compounds **B2** and **B3**; however, its effect on the expression of BCL-2, an antiapoptotic protein, was not detected. The activation of caspase-3, a common protein involved in both the intrinsic and extrinsic apoptotic pathways, induced by both compounds, has also been demonstrated [26].

The bark of trees of the genus *Tabebuia* is rich in bioactive compounds, and the most studied compound is lapachol, a 1,4-naphthoquinone. Zu et al. [33] evaluated the potential of lapachol to inhibit the RSK2 protein (ribosomal protein S6 kinase 2). RSK2 is a kinase that is activated via mitogen-activated protein kinase (MAPK) in response to growth factors such as epidermal growth factor receptor (EGFR). RSK2 inhibitors suppress cell proliferation and induce apoptosis in cancer cells. When epidermal growth factor (EGF) is blocked in resistant cancer cells, RSK2 is activated, which is important for cell survival. In this study, high levels of RSK2 expression were observed during the proliferation of esophageal squamous cell carcinoma (ESCC) cells, and these levels decreased when RSK2 was silenced, highlighting the role of RSK2 in ESCC cell proliferation. Treatment with lapachol, which is isolated from *Tabebuia avellanedae*, inhibited RSK2 (with an IC_50_ of 2 μM) and induced alterations in the activation of transcription factors important for protein production, specifically cAMP-responsive element binding protein (CREB) and activating transcription factor 1 (ATF-1), which are involved in proliferation and cell survival processes. Furthermore, the inhibition of ESCC cell growth by lapachol depends on the presence of RSK2 in the cells, indicating that its mechanism of action is limited to binding with RSK2. Additionally, the effects of lapachol on cell-cycle-related proteins were evaluated, and lapachol strongly inhibited the expression of cyclin B1, cyclin D3, and cyclin D1, as well as the phosphorylation of CDK2, and attenuated the phosphorylation of histone H3, which is located directly downstream of RSK2. Treatment with lapachol induces apoptosis in ESCC via the intrinsic pathway by increasing the expression of proteins such as caspases 3 and 7, cleaved PARP, cytochrome c, and Bax [33].

**Figure 4:**
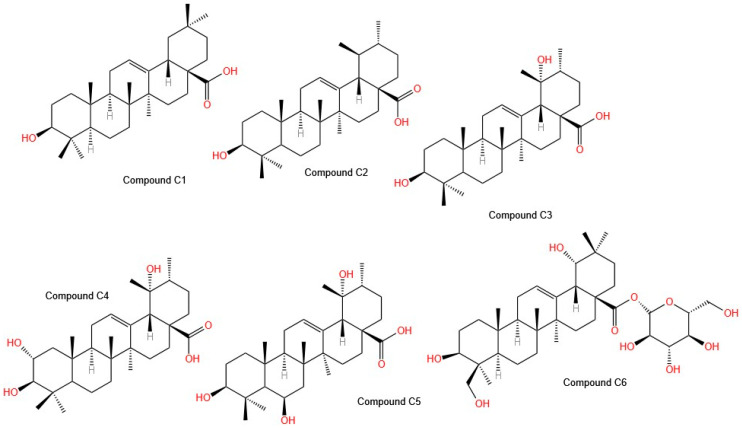
Chemical structures of triterpenes and isolated Compounds **C1**–**C6**. Figure created with KingDraw, 2018–2025.

Mahmoud et al. evaluated the total extract, subfractions, and isolated compounds of *Tabebuia aurea*. The ethyl acetate fraction showed great potency against the three evaluated cell lines, A549 (lung cancer), MCF-7 (breast cancer), and HepG2 (hepatocarcinoma), with IC_50_ values of 34.2 ± 3.5, 38.2 ± 2.8, and 42.3 ± 2.9 µg/mL, respectively. These values were very similar to those obtained with the positive control etoposide, a semisynthetic chemotherapeutic drug (IC_50_ values for each line: 28.1 ± 4.2, 22.5 ± 4.5, and 20.4 ± 0.8 µg/mL, respectively). The use of reversed-phase high-resolution liquid chromatography (RP-HPLC) led to the isolation of six compounds (Figure 4), such as **C1**: oleanolic acid; **C2**: ursolic acid; **C3**: pomolic acid; **C4**: tormentic acid; **C5**: 3 β, 6 β, 19 α-trihydroxy-urs-12-en-28-oic acid; and **C6:** spathodic acid 28-O-β-D-glucopyranoside, which were identified via spectroscopic techniques. In terms of selectivity, Compound **C2** is selective for the HepG2 cell line, with an IC_50_ of 26 µg/mL, whereas Compound **C4** is selective for the MCF-7 cell line, with an IC_50_ of 25.3 µg/mL; however, Compound **C1** is less selective [35]. These isolated molecules are of great interest for further studies to determine their mechanisms of action. Importantly, Compound **C5** was isolated for the first time from *Tabebuia aurea*, Compounds **C3** and **C4** were isolated for the first time from the genus *Tabebuia,* and Compound **C6** was isolated for the first time from the family *Bignoniaceae* [34].

The other isolated molecules analyzed included polysaccharides and extracts from the bark of two species, *Handroanthus heptaphyllus* and *Handroanthus albus*. Carlotto et al. proposed several techniques to elucidate soluble polysaccharide fractions, including monosaccharide analysis, methylation analysis, high-performance size-exclusion chromatography (HPSEC), and nuclear magnetic resonance spectroscopy (NMR). The fractions comprised HHBSF (*H. heptaphyllus* bark—crude polysaccharide) and HABSF (*H. albus* bark—crude polysaccharide). The first fraction exhibited a retention time of 55.5 min, corresponding to a molecular weight of 8.9 kDa. The second fraction displayed a heterogeneous elution profile with two distinct peaks: 53.7 min/16.6 kDa and 56.5 min/6.3 kDa. Glycosidic linkage analysis by methylation demonstrated the presence of many partially O-methylated alditol acetate derivatives. NMR analysis revealed the presence of galactoglucomannan, type II arabinogalactan (AGII), and type I rhamnogalacturonan (RGI) in both fractions; however, only HABSF significantly inhibited the growth of MCF-7 (CC_50_ = 327 μg/mL) and Caco-2 (CC_50_ = 2258 μg/mL) cells. Differences in the fine structure and proportion of their polysaccharides and possibly, in the composition of associated phenolic compounds, could explain the different effects of HHBSF and HABSF. To determine the selectivity of the HASBF fraction, its cytotoxic effect was evaluated in Vero cells (normal monkey kidney cells), with 95% viability at a concentration of 327 µg/mL [35].

The antiproliferative potential of β-lapachone has been widely studied; however, few studies have addressed the issue of selectivity, that is, the ability of a compound to affect only tumor cells without harming healthy cells. Among all the studies evaluating isolated molecules, only one study from Lima et al. presented results related to their selectivity [36]. In this study, the IC_50_ values of β-lapachone in the ACP02 (gastric adenocarcinoma), MCF-7, HCT116 (colon cancer), and HEPG2 cell lines were 3.0, 2.2, 1.9, and 1.8 μg/mL, respectively. In addition, the authors also evaluated the safety profile of β-lapachone [36]. In their study, Lima et al. performed a comet assay on HepG2 cells to evaluate genotoxicity, observing significant DNA damage after treatment with 1.5 µg/mL β-lapachone, a result comparable to that of the positive control, doxorubicin. The authors suggested that this damage may be related to the generation of reactive oxygen species (ROS), which is mediated by the NQO1 enzyme, a mechanism extensively studied by Lamberti et al. [10]. However, the latter group did not perform selectivity tests. Lima et al. warned about the risks of using β-lapachone as a therapeutic agent, as healthy cells expressing NQO1 could also suffer the same cytotoxic effect. Furthermore, even at low concentrations, β-lapachone exerts genotoxic effects, which do not induce immediate apoptosis but can cause mutations in exposed cells. For this reason, they recommend developing targeted delivery strategies, such as those utilizing nanotechnology or structural modifications of the compound, to minimize adverse effects on nontumor cells [36].

Notably, in terms of plant extracts with antiproliferative activity, Suseela et al. [13] proposed the use of silver nanoparticles, which showed enhanced activity when combined with a plant extract. However, this study also lacks a selectivity analysis, highlighting the persistence of a gap in the scientific literature regarding the use of natural compounds in cancer therapies.

The reviewed studies highlight the antitumor potential of compounds isolated from *Tabebuia* species, demonstrating various mechanisms of action, including the inhibition of cell proliferation, the induction of apoptosis, and the alteration of key signaling pathways. The C9-type iridoid [2], argenteoside A, which is isolated from *T. argentea*, strongly inhibits the ATPase activity of HSP90, a chaperone essential for protein folding in cancer cells. The stable binding of argenteoside A with HSP90 suggests a potential mechanism for destabilizing essential proteins and inhibiting tumor growth. Low KD and Kd values also indicate high binding affinity, reinforcing its therapeutic relevance. However, further studies are needed to evaluate its selectivity index and its effects on normal cells.

The flavonoid trimethoxyflavone (TMF) [5], which is extracted from *T. chrysantha*, exhibits antiangiogenic activity by suppressing the STAT3-MMP9 signaling pathway, a crucial pathway for tumor vascularization. TMF was observed to reduce ERK activation, limiting angiogenesis, suggesting that natural polyphenols may act as inhibitors of tumor progression.

β-lapachone, a widely studied naphthoquinone, displays strong antitumor activity. It was found to suppress telomerase activity and negatively regulate the transcription factor Sp1 [30], a key factor in tumor survival. β-lapachone reduces tumor proliferation by inducing cell cycle arrest in the G2/M phase [9]. It also induces apoptosis through both intrinsic and extrinsic pathways, which are mediated by the activation of caspases 3, 8, and 9.

The effectiveness of β-lapachone in human malignant melanoma (G361 and SK-MEL-28) and colorectal cancer (CT26) [31] underscores its potential across multiple cancer types. Additionally, its ability to modulate metalloproteinases (MMPs) and epithelial–mesenchymal transition (EMT) markers suggests antimetastatic properties, crucial factors in cancer therapy.

The mechanism of action of β-lapachone depends on NQO1 expression [10], an oxidoreductase that enhances its cytotoxic effect on cancer cells. Studies suggest that combination therapies with NQO1 inducers, such as sulindac or photodynamic therapy (PDT), can increase its efficacy, especially in lung cancer cells with low NQO1 expression. The synergistic strategy of β-lapachone and PDT increased tumor sensitivity to treatment, while reducing systemic toxicity.

Lapachol, another widely studied naphthoquinone, has shown potential as an RSK2 inhibitor, a kinase activated by MAPK in response to growth factors such as EGFR [33]. The inhibition of RSK2 by lapachol reduced cell proliferation and affected the expression of proteins involved in tumor survival, suggesting its potential use as a targeted therapy for esophageal squamous carcinoma (ESCC).

Other isolated molecules, including triterpenes [34] and polysaccharides [35], have also been evaluated for oncological therapies. A study revealed that triterpenes isolated from T. aurea exhibited strong cytotoxicity against lung, breast, and liver cancer, with some selectivity for MCF-7 and HepG2 cells. Notably, three triterpenes previously unreported in *Tabebuia* were identified, opening new opportunities for drug development. On the other hand, polysaccharides from *Handroanthus heptaphyllus* and *H. albus* exhibited selective cytotoxicity against MCF-7 and Caco-2 cells, suggesting a potential role in modulating the tumor microenvironment.

### 2.4. Synthesized Molecules with Anti-Proliferative Activity

**Figure 5:**
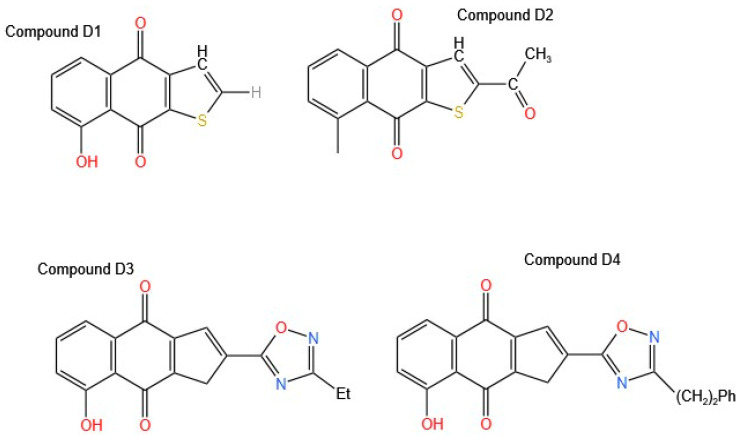
Chemical structures of synthesized compounds **D1**–**D4**. Figure created with KingDraw, 2018–2025.

Based on findings regarding the anti-proliferative activity of extracts and isolated molecules from *Tabebuia* species, some studies have focused on analyzing how synthesized molecules or modifications to the molecules identified and isolated from the extracts can increase their activity and allow for the discovery of new molecules with antitumor potential. In this context, few studies have been conducted on the genus *Tabebuia* (Table 4). Bannwitz et al. [14] analyzed 71 analogs that modify the basic structure of naphtho [2,3-b] furan-4,9-dione; in this way, they analyzed how, by varying the functional groups and their position in the molecule, the results of antiproliferative activity were obtained in HaCaT keratinocytes by performing structure–activity relationship (SAR) analysis. As a result of all these modifications, three promising Compounds (**D1**, **D2**, and **D3**, Figure 5) were obtained, proving to be powerful generators of free radicals.

Compound **D1** corresponded to the basic structure of the molecule. Compound **D2** was modified with an acetyl group at position 2, which resulted in better activity than that observed when it was modified with a hydroxy group, and Compound **D3** was modified with an oxadiazole. The release of lactate dehydrogenase (LDH) from the culture supernatant was determined. The release of LDH is commonly used as an indicator of plasma membrane damage. In addition, apoptosis was measured by 7-aminoactinomycin D (7-AAD) staining, and the ability of selected 8-hydroxynaphtho [2,3-b] thiophene-4,9-diones to stimulate superoxide generation was determined. Compounds **D2** and **D3** generated significant amounts of superoxide, and these results are attributed to the substitutions at position 2. Compounds **D3** and **D4**, which are oxadiazoles, exhibited IC_50_ values of 0.19 and 0.48 µM, respectively. Compound **D3**, which exerts a potent antiproliferative effect on keratinocytes, is of significant interest for the treatment of pathologies such as psoriasis and malignant conditions associated with these cells, including basal cell carcinoma (BCC) and cutaneous squamous cell carcinoma (cSCC). Potent inhibitors of keratinocyte hyperproliferation, with electron-withdrawing functionalities, e.g., acyl, carboxylic acids, and carboxamide, or their bioisosteric replacements, with a 1,2,4-oxadiazole ring at position 2 of the tricyclic system, were obtained. Compared with the control, all lapacho analogs (5 μM/L) significantly increased early apoptosis, with the most significant effect observed with the oxadiazoles. However, the amount of superoxide produced by lapacho quinones does not correlate with their ability to induce cell death [14].

Quinones are highly redox-active molecules that can engage in redox cycles with their semiquinone radicals, forming ROS, including superoxide, hydrogen peroxide, and ultimately, the hydroxyl radical. The production of ROS can cause severe oxidative stress within cells by forming oxidized cellular macromolecules, including lipids, proteins, and DNA. Eyong et al. [37] prepared 45 new derivatives of lapachol that are structurally related to β-lapachone, α-lapachone, and 2-acetylfuronaphtoquinone, with potentially enhanced biological activity. They tried to remove the quinone groups (orthoquinones) to yield indane carboxylic acids via benzilic acid rearrangement and phenazines via a Schiff base reaction to avoid ROS production associated with drugs that possess a quinone moiety.

**Figure 6:**
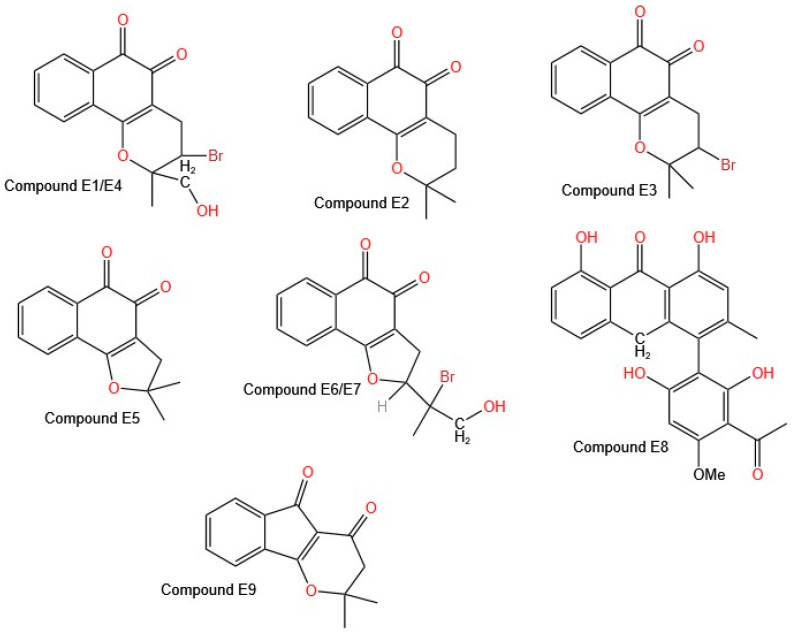
Chemical structures of the derivatives of β-lapachone, α-lapachone, and 2-acetylfuranaphthoquinone, Compounds **E1**–**E9**. Figure created with KingDraw, 2018–2025.

All the synthesized compounds showed cytotoxic activity against all the cell lines evaluated (neuroblastoma, melanoma, glioblastoma, and non-small cell lung cancer) at a concentration of 10 µM. Only Compound **E1** (Figure 6 shows the structures of molecules **E1**–**E9**), derived from β-lapachone, showed no activity against the neuroblastoma cell lines (BE (2)-C, Kelly, SKNSH, and CHLA-90).

Eyong et al. reported that fourteen of the tested compounds were most active, with submicromolar to low micromolar IC_50_ values against the A549 (non-small cell lung cancer), SKMEL-28 (melanoma), and U373 (glioma) cell lines. Among these compounds, seven are based on β-naphthoquinone, and five are based on the α-naphthoquinone [2,3-b] furan structural skeleton [37]. The best activity was found for compounds in which the β-naphthoquinone ring was fused with a saturated fraction of pyran (Compounds **E1**, **E2**, **E3**, and **E4**) or furan (Compounds **E5**, **E6**, and **E7**). The derivative of the class α-naphthoquinone [2,3-b] furan, modified with an acetyl furan group, showed the highest cytotoxic activity, which was attributed to the carbonyl group of the acetoxy in the furan ring. Compound **E8** shows an IC_50_ value close to 1 µM; it is anthrone in nature, and the literature reports that this type of compound induces necrotic cell death in cancer cells, identifying it as a promising anticancer agent for combating cancers with intrinsic resistance to apoptosis [38,39]. In addition, a new indeno [1,2-b] pyran derivative (E9) exhibited potent cytotoxic activity, with IC_50_ values in the micromolar range for the A549 and SKMEL-28 cell lines [36].

Previous reports on the potential activity of naphthoquinones and their presence in species of the genus *Tabebuia* led to the synthesis of these molecules and the evaluation of their potential. Inagaki et al. [40] based their synthesis on previous reports but modified the catalysts used, increasing the percentage of yield of the reactions from 20% to 56% with the use of palladium(II) acetate (Pd(OAc)_2_) and cuprous bromide (CuBr). The objective was to synthesize naphthoquinones in which the benzene ring was substituted with a methoxy group. Inagaki et al. reported that modifications to these molecules improve their anticancer activity.

**Table 4:** Data extraction of synthesized molecules with antiproliferative activity.

Reference	Cell Line	Biological Activity	Biological Response Method	Number of Compounds Evaluated	IC_50_
[14]	HaCat	Antiproliferative Apoptosis	LDH	**71**	No
[37]	A549 SKMEL-28 U373	Antiproliferative	MTT	**45**	No
[40]	HL-60 U937	Cytotoxic	MTT	**20**	Yes

IC_50_: 50% inhibitory concentration; LDH: lactate dehydrogenase; MTT: 3-(4,5-dimethylthiazolyl-2)-2,5-diphenyltetrazolium bromide.

**Figure 7:**
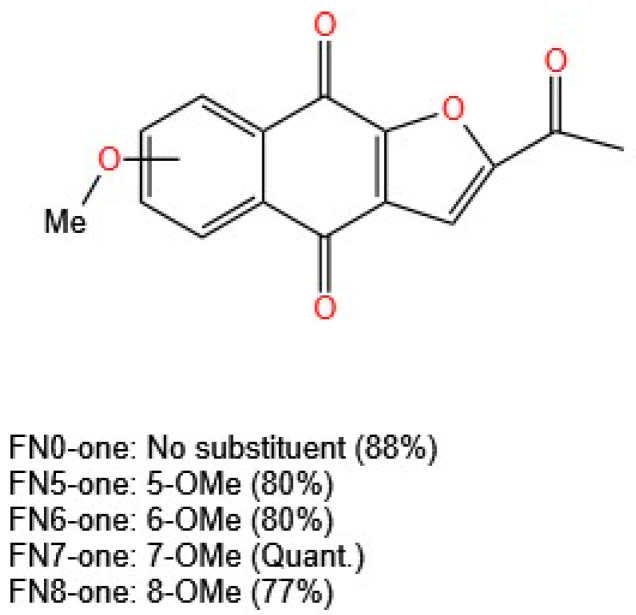
Structures of the FN0-one and FN5-8-one types. Figure created with KingDraw 2018–2025.

The in vitro cytotoxicity of these compounds was evaluated via the MTT assay in U937 (human histiocytic lymphoma) and HL-60 (human acute promyelocytic leukemia) cells. All the compounds inhibited cell proliferation in a concentration-dependent manner. The activity was weak for those analogs with a methoxy group at position 5 or 8. The use of cisplatin as a control resulted in an IC_50_ value of 1.9 µM in HL-60 cells, in contrast to the results for the three furanonaphthoquinones evaluated, with IC_50_ values of 0.58, 0.87, and 0.59 µM for 2-acetylnaphtho [2,3-b] furan-4,9-dione (FN0-one, no substituents), FN6-one (6-OMe substituted), and FN7-one (7-OMe substituted), respectively (Figure 7). This report is concise in its presentation of information, stating only that this activity remains related to the redox cycle and the generation of free radicals [40]. The mechanism of action of naphthoquinones and their derivatives as antiproliferative agents has been studied in several studies using different cell lines. The mechanisms can be associated with the generation of ROS, DNA fragmentation, induction of apoptosis, alterations in the mitochondrial membrane potential, inhibition of topoisomerases, cell cycle arrest, alteration of MAPK pathways, and STAT3 inhibition [41,42].

The analysis of the synthesized molecules revealed that synthetic modifications of naphthoquinones and their related compounds derived from *Tabebuia* can increase their antiproliferative activity, allowing for the development of new anticancer agents. Researchers have identified more effective and selective derivatives by modifying functional groups and molecular structures.

Bannwitz et al. [14] analyzed 71 naphtho[2,3-b] furan-4,9-dione analogs to evaluate how structural modifications influence antiproliferative activity in HaCaT keratinocytes. SAR analysis revealed three promising Compounds (**D1**, **D2**, and **D3**) that act as potent free radical generators. Compound **D2**, modified with an acetyl group at position 2, showed higher activity than that modified with a hydroxyl group. Compound **D3**, with an oxadiazole substitution, significantly increased superoxide production and induced apoptosis, with IC50 values of 0.19 and 0.48 µM, respectively. The ability of lapachone analogs to induce apoptosis is correlated with the presence of electron-withdrawing functional groups, such as acyls, carboxylic acids, and carboxamides.

Eyong et al. [37] synthesized 45 new derivatives of lapachol, β-lapachone, α-lapachone, and 2-acetylfuranonaphthoquinone, aiming to reduce ROS-associated toxicity while maintaining their anticancer properties. Fourteen compounds exhibited strong cytotoxicity, with IC_50_ values ranging from submicromolar to low micromolar values against A549 (non-small cell lung cancer), SKMEL-28 (melanoma), and U373 (glioma) cells. The highest activity was observed in compounds in which the β-naphthoquinone ring was fused with a pyran (**E1**–**E4**) or furan (**E5**–**E7**). Additionally, Compound **E8**, an anthrone-based compound, exhibited an IC_50_ of approximately 1 µM and induced necrotic cell death, making it a promising candidate for treating cancers resistant to apoptosis. Compound **E9**, an indeno [1,2-b] pyran derivative, showed potent cytotoxic activity in the A549 and SKMEL-28 cell lines, reinforcing the importance of structural modifications in improving anticancer activity.

Inagaki et al. [40] synthesized naphthoquinones substituted with methoxy groups on the benzene ring, optimizing the reaction conditions and increasing yields from 20% to 56% using palladium(II) acetate (Pd(OAc)_2_) and cuprous bromide (CuBr) as catalysts. Their study revealed that derivatives with methoxy substituents at positions 5 or 8 presented weak activity. Unsubstituted furanonaphthoquinones (FN0-one) and those substituted at positions 6 or 7 (FN6-one and FN7-one) showed strong activity, with IC_50_ values of 0.58, 0.87, and 0.59 µM in HL-60 leukemia cells, surpassing the results for the control drug cisplatin (IC_50_ = 1.9 µM). The mechanism of action remains closely linked to the redox cycle and free radical generation, underscoring the need for further studies to assess long-term effects and toxicity.

Importantly, within the *Bignoniaceae* family, the genus *Tabebuia* has been the most extensively studied from the perspective of its biological activities, and the most studied species are *Tabebuia avellanedae* (Pau d’arco), *Tabebuia argentea*, *Tabebuia rosea*, *Tabebuia aurea*, *Handroanthus impetiginosus*, *Handroanthus serratifolius*, *Handroanthus chrysanthus*, *Handroanthus chrysotrichus*, and *Handroanthus ochraceus*. A complete review of the species and biological activities evaluated, as well as the molecules isolated from these species, is beyond the scope of this paper; however, such information can be found in reviews published recently by Hamed and El-Hawary [43,44].

## 3. Materials and Methods

### 3.1. Search Strategy

A review of articles concerning the antiproliferative and antitumor effects of plant extracts and isolated and synthesized molecules was carried out in three bibliographic databases: PubMed, Scopus, and SciELO. The following terms were used: (“tabebuia” AND “antineoplastic agents”/“neoplasms”/“cancer”/“antiproliferative”) AND (“*Bignoniaceae*” AND “antineoplastic agents”/“neoplasms”/“cancer”/“antiproliferative”) AND (“chemotherapeutic”). The publication dates ranged from January 2013 to December 2024. Importantly, as we used MeSH terms for the search, *Handroanthus* was included in the term *Tabebuia*.

### 3.2. Study Selection

Original research articles written in Spanish, English, or Portuguese presenting the results of studies on the half-maximal inhibitory concentration (IC_50_), half-cytotoxic concentration (CC_50_), and phytochemical characterization of extracts in vitro, in vivo, or in silico were considered. Reviews, conference articles, proceedings, theses, editorials/letters, patents, case reports, and analyses of extracts without phytochemical characterization were excluded from this systematic review. Once the search strategy and inclusion criteria were established (Table 4), the total number of results from each database was counted before and after applying the publication date filter. These were imported and saved in EndNote X7 software (Thomson Reuters, Culver City, CA, USA), where duplicate references were identified and eliminated via the software’s built-in features and excluded from this review. The full texts of the remaining articles were read to select those that met the inclusion criteria.

### 3.3. Assessment of Methodological Quality

**Figure 8:**
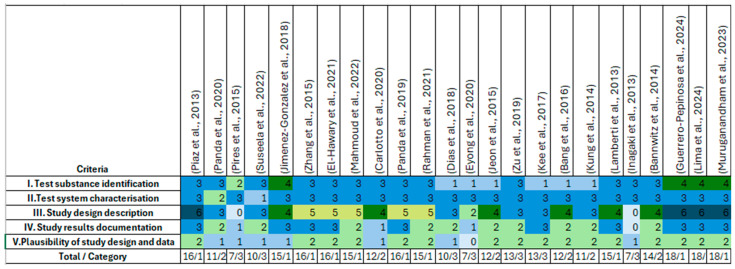
The figure presents the total sum for each criterion evaluated. Ultimately, the total score indicates the category of each item: Category 1: reliable without restrictions (15–18 points). Category 2: reliable with restrictions (11–14 points). Category 3: unreliable (less than 11 points). The colors indicate the category for each item, [2,5,6,7,8,9,10,11,13,14,22,24,26,28,29,30,31,32,33,34,35,36,37,40].

All included studies were evaluated using ToxRTool V1.0, a software-based tool developed by the European Commission’s Joint Research Center in 2009 to provide comprehensive criteria and guidance for evaluating the inherent quality of toxicological data [45]. This approach applies to various types of experimental data, including in vitro and in vivo studies. The tool comprises a list of 21 criteria for in vivo studies and 18 criteria for in vitro studies. Each criterion is assigned either a “1” (one point) or a “0” (zero points). The ToxRTool includes five groups of criteria, in both cases, to evaluate the studies: (i) test substance identification, (ii) test system characterization, (iii) study design description, (iv) study result documentation, and (v) plausibility of the study design and results. The selected articles were assessed via in vivo and in vitro spreadsheets from the ToxRTool to evaluate the quality of the studies. The results are summarized in Figure 8.

## 4. Conclusions

A review of studies on the antiproliferative activity of *Tabebuia* species confirms their therapeutic potential as a source of new antitumor agents. Extracts obtained from leaves and bark, as well as the isolated and synthesized molecules, have demonstrated cytotoxic and apoptotic effects in various tumor cell lines. These results suggest that the antitumor effects depend not only on the specific metabolites present in the extracts but also on the synergy between these compounds, which enables the modulation of toxicity and enhanced tolerability in the body.

From a future perspective, this review highlights the importance of ongoing studies that assess selectivity and long-term toxicity to minimize adverse effects on normal cells. In vivo studies should also be conducted to better understand the biodistribution and pharmacokinetics of these compounds, as well as to optimize the synthesis of *Tabebuia*-derived molecules to increase their efficacy. The use of NQO1 inducers in combination with β-lapachone has demonstrated the importance of combination therapeutic strategies, highlighting their potential in cancer therapy.

In this context, this review provides an updated overview of the potential of Tabebuia in oncology, laying the foundation for the design of new studies to validate these compounds in preclinical and clinical models. Thus, research on natural compounds and their derivatives remains a key strategy for developing more effective and less toxic treatments in the fight against cancer.

## Figures and Tables

**Table 1:** Inclusion and exclusion criteria used in this systematic review.

Parameter	Inclusion Criteria	Exclusion Criteria
1. Language	English, Spanish, and Portuguese	Any other language
2. Type of publication	Original articles	Systematic reviews, gray literature, or any other type of publication
3. Type of study	Biological activity, in vitro, in vivo, and clinical studies	Any other type of study
4. Phytochemical characterization	Articles where extracts were evaluated, and their phytochemical characterization was performed	Articles where extracts were evaluated, and the phytochemical characterization was not performed
5. Biological activity evaluation	Antiproliferative	Any other activity

**Table 2:** Data extraction of plant extracts with antiproliferative activity.

Reference	Species	Cell Line	Biological Activity	Biological Response Method	Part of the Plant Used	Solvent Used	CC_50_	IC_50_	SI	Other Biological Activities Evaluated
[11]	*T. rosea*	HEK-293, HepG2, HeLa, B16F10, MCF7	Cytotoxic—antiproliferative	MTT	Leaves and stem	n-Hexane, chloroform, ethyl acetate	X	X	X	None
[7]	*T. impetiginosa*	MCF-7, NCI-H460, HeLa, HepG2	Cytotoxic	Sulforhodamine B	Inner bark	Methanol		X	X	Antioxidant
[13]	*T. rosea alba*	A549	Cytotoxic Apoptosis	MTT	Leaves	Ethanol		X		Mitochondrial membrane potential
[6]	*T. pallida*, *T. pulcherrima*, *T. rosea*, *T. argentea*, *T. guayacan*	HepG2, MCF-7, CACO2	Cytotoxic	MTT	Leaves and stem			X		None
[28]	*T. chrysantha*	EAC	Cytotoxic Apoptosis	MTT	Stems	Methanol		X		None
[24]	*T. pallida*	AC	Cytotoxic Apoptosis	MTT	Leaves Stems Fruits	Methanol		X		Antitumoral, antioxidant, apoptosis (in vitro, in vivo, and in silico)
[22]	*T. rosea*	THP-1, Jurkat, and PBMC	Apoptosis	MTT	Inner bark	n-butanol		X	X	Mitochondrial membrane potential
[29]	*T. rosea*	L929	Cytotoxic	MTT	Seeds	Water		X		DPPH

CC_50_: 50% cytotoxic concentration; IC_50_: 50% inhibitory concentration; SI: selectivity index; MTT: (3-(4,5-dimethylthiazolyl-2)-2,5-diphenyltetrazolium bromide) assay; PBMC: peripheral blood mononuclear cell; DPPH: 2,2-diphenyl-1-picrylhydrazyl assay.

**Table 3:** Data extraction of isolated molecules with antiproliferative activity.

Reference	Species	Cell Line	Biological Activity	Biological Response Method	Genes/Proteins Evaluated	Number of Compounds Evaluated	Part of the Plant Used	Source	IC_50_	SI	Other Biological Activities Evaluated
[2]	*T. argentea*	HeLa	Cytotoxic Antiproliferative	MTT	HSP90	**24**	Leaves	Natural	Yes	No	Citrate synthase
[5]	*T. chrysantha*	EAC	Antiproliferative	MTT	STAT 3-MMP9/VEGF-A	**4**	Stem	Natural	No	No	None
[30]	*T. avellanedae*	HN22 HSC4	Antiproliferative Apoptosis	MTT	Sp1 P27 P21 Cyclin D1 Survivin	**1**	Stem bark	Natural	No	No	None
[33]	*T. avellanedae*	KYSE30 KYSE450 KYSE510 ESCC	Apoptosis	MTT	RSK2	**1**	Stem bark	Natural	Yes	No	None
[31]	*T. impetiginosa*	CT26 MC38	Apoptosis	WST-8	None	**1**	Stem bark	Natural	No	No	None
[8]	*T. avellanedae*	G361 SK-MEL-28	Antiproliferative Apoptosis	MTS	Sp1	**1**	Stem bark	Natural	No	No	Mitochondrial membrane potential
[9]	*T. avellanedae*	OSCC human (HSC3, SCC4, SCC9, SCC15, SCC25)	Cytotoxic Apoptosis	Alamar blue	None	**3**	Stem bark	Natural	Yes	Yes	Mitochondrial membrane potential; cytotoxic activity in spheroids and fibroblasts in xenografts.
[26]	*T. avellanedae*	A549 MCF-7	Apoptosis	MTT	None	**3**	Stem bark	Natural	No	No	None
[32]	*T. avellanedae*	CL1-1 CL1-5	Cytotoxic	MTT	NQO1	**1**	Stem bark	Natural	No	No	Intracellular calcium concentration
[10]	*T. avellanedae*	MCF-7c3	Cytotoxic	MTT	None	**1**	Stem bark	Natural	Yes	No	None
[35]	*H. heptaphyllus* *H. albus*	Caco-2 MCF-7	Cytotoxic	MTT	None	Two polysaccharide fractions	Bark		No	No	None
[34]	*T. aurea*	A549 MCF-7 HepG2	Cytotoxic	MTT	None	**6**	Leaves	Natural	Yes	No	None
[22]	*T. rosea*	THP-1, Jurkat and PBMC	Apoptosis	MTT	Bax, p38	**2**	Inner bark	Natural	Yes	Yes	None
[36]	*Tabebuia* spp.	ACP02, MCF7, HCT116, HEPG2	Apoptosis	MTT	None	**1**	Sawdust of Ipê wood	Natural	Yes	No	None

IC_50_: 50% inhibitory concentration, SI: selectivity index, MTT: (3-(4,5-dimethylthiazolyl-2)-2,5-diphenyltetrazolium bromide) assay, WST-8: (2-(2-methoxy-4-nitrophenyl)-3H-nitrophenyl)-5-(2,4-disulfophenyl)-2H-tetrazolium, monosodium salt), MTS: (3-(4,5-dimethylthiazol-2-yl)-5-(3-carboxymethoxyphenyl)-2-(4-sulfophenyl)-2H-tetrazolium), HSP90: heat shock protein 90, STAT 3: signal transducer and activator of transcription 3, MMP9: matrix metalloproteinase 9, VEGF-A: vascular endothelial growth factor A, Sp1: specificity protein 1, P27: P27 protein, P21: cyclin-dependent kinase inhibitor p21, RSK2: ribosomal S6 kinase 2, and NQO1: NAD(P)H quinone oxidoreductase 1.

## Data Availability

The original contributions presented in the study are included in the article, and further inquiries can be directed to the corresponding author.
